# Four new members of the family *Cytophagaceae*: *Chryseosolibacter histidini* gen. nov., sp. nov., *Chryseosolibacter indicus* gen. nov., sp. nov., *Dawidia cretensis*, gen. nov., sp. nov., and *Dawidia soli,* gen. nov., sp. nov. isolated from diverse habitat

**DOI:** 10.1007/s10482-022-01756-2

**Published:** 2022-06-22

**Authors:** Senlie Octaviana, Stefan Lorenczyk, Frederike Ackert, Linda Fenske, Joachim Wink

**Affiliations:** 1 Microbial Strain Collection, Helmholtz Center for Infection Research, Inhoffenstraße 7, 38124 Brunswick, Germany; 2 Research Center for Applied Microbiology, National Research and Innovation Agency (BRIN), Cibinong, 16911 Indonesia; 3 Faculty of Mechanical Engineering, Technical University of Braunschweig, Schleinitzstraße 20, 38106 Brunswick, Germany; 4 Bioinformatic and Systems Biology, Justus Liebig University Gießen, Heinrich-Buff-Ring 58, 35390 Gießen, Germany

**Keywords:** Cytophagaceae, Diverse habitat, Gliding bacteria

## Abstract

Four novel strains were isolated: PWU4^T^ and PWU20^T^ were both from soil in Germany, PWU5^T^ was isolated from soil in India and PWU37^T^ was obtained from sheep faeces collected on the Island of Crete. Cells of each were observed to be Gram-negative, strictly aerobic, rod shaped, and to grow optimally between 28 and 34 °C, between pH 7.0 and 8.0 and without the addition of NaCl. The strains were found to be catalase and oxidase-negative and able to grow on most mono- and disaccharides, a few polysaccharides and organic acids. Their predominant menaquinone was identified as MK-7. Their major fatty acids were identified as C_16:1_ *ω*7*c* (PWU4^T^ and PWU20^T^) and C_16:1_ *ω*5*c* (PWU5^T^ and PWU37^T^). The DNA G + C contents of strains PWU4^T^, PWU20^T^, PWU5^T^ and PWU37^T^ were determined to be 50.2 mol%, 51.6 mol %, 39.8 mol% and 53.8 mol%, respectively. The 16S rRNA gene sequence analysis revealed that the close relatives *Ohtaekwangia koreensis* 3B-2^T^ and *Ohtaekwangia kribbensis* 10AO^T^ share less than 93.8% sequence similarity. The strains were classified in two groups, where PWU4^T^ and PWU20^T^ share 93.0% sequence similarity, and PWU5^T^ and PWU37^T^ share 97.5% sequence similarity. However, the members of each group were concluded to represent different species based on the low average nucleotide identity (ANI) of their genomes, 69.7% and 83.8%, respectively. We propose that the four strains represent four novel species of two new genera in the family *Cytophagaceae*. The type species of the novel genus *Chryseosolibacter* is *Chryseosolibacter histidini* gen. nov., sp. nov. with the type strain PWU4^T^ (= DSM 111594^T^ = NCCB 100798^T^), whilst strain PWU20^T^ (= DSM 111597^T^ = NCCB 100800^T^) is the type strain of a second species, *Chryseosolibacter indicus* sp. nov. The type species of the novel genus *Dawidia* is *Dawidia cretensis* gen. nov., sp. nov. with the type strain PWU5^T^ (= DSM 111596^T^ = NCCB 100799^T^), whilst strain PWU37^T^ (= DSM 111595^T^ = NCCB 100801^T^) is the type stain of a second species, *Dawidia soli* sp. nov.

## Introduction

The family *Cytophagaceae* was originally introduced in 1940 by Stanier (Stanier 1940) and it is the largest family within the order *Cytophagales* (Albers and Siebers 2014). Isolates of the family *Cytophagaceae* are Gram-stain negative chemoorganotrophic aerobic bacteria, but also a few anaerobes (Nakagawa 2011). Furthermore, they are widely distributed in nature such as soil (Hirsch et al. 1998; Kim et al. 2013; Zhang et al. 2009), freshwater (Baik et al. 2006; Maejima et al. 2020), airborne (Buczolits et al. 2002), dessert (Zhou et al. 2007) and a glacier field (Chaturvedi et al. 2005).

Since the ‘golden age’ of antibiotic discovery, members of the phylum *Bacteriodetes* including the classes *Flavobacteriia* and *Cytophagia*, have contributed as producers of antimicrobial bioactive compounds (Ikegami et al. 1990; Katayama et al. 1985; Okanya et al. 2011a, b; Park et al. 2008; Singh et al. 1982). During the screening of antimicrobial activity from members of the Reichenbach culture collection held at the Helmholtz Center for Infection Research (HZI) Germany, four novel bacterial strains (designated PWU4^T^, PWU5^T^, PWU20^T^ and PWU37^T^) were identified. The aim of the present study was to explore the taxonomic status of the four bacteria as novel species by using a polyphasic approach.

## Material and methods

### Isolation of bacterial strains and culture conditions

Strains PWU4^T^, PWU20^T^ and PWU37^T^ were isolated from soil samples collected in May 1990 at Braunschweig, Germany (52.22090N 10.50902E, PWU4^T^); in May 1989 at Lucknow, Uttar Pradesh, India (26.8684N 80.90979E, PWU20^T^); in September 1991 at Braunschweig (52.21501N 10.53329E, PWU37^T^); strain PWU5^T^ was isolated from sheep faeces with plant residues collected in July 1988 at Crete Island (35.2463N 25.09705E). The strains were isolated using a dilution method on agar plates following the protocol of Reichenbach (Reichenbach 1992), maintained in E medium and kept in this medium at − 80 °C for long-term preservation. *Ohtaekwangia koreensis* 3B-2^T^ KCTC23018^T^ and *Ohtaekwangia kribbensis* 10AO^T^ KCTC23019^T^, which were isolated by Yoon et al. (2011a), were used as references strains and were grown under the same culture conditions.

### Morphological, physiological and chemotaxonomy analysis

Phenotypic characterisation was performed following the protocols described previously (Kim et al. 2013; Maejima et al. 2020; Yoon et al. 2011a). Morphological characteristics of the strains were observed using light microscopy (Zeiss Axio Scope A1. Microscope) with Axio-Vision Rel. 4.8 software. For physiological and chemotaxonomic tests, the four strains were grown without NaCl in E broth medium at their optimum pH and temperature of pH 7 and 30 °C for strain PWU4^T^, pH 7 and 28 °C for strains PWU20^T^ and PWU5^T^, pH 7.4–8.0 and 34 °C for strain PWU37^T^. Growth at various temperatures, pH and NaCl concentrations was carried out aerobically on E agar medium. To determine the optimal temperature and pH for growth, duplicate plates were incubated at 4–44 °C and also at pH 5.0–9.5 as described previously (Mohr et al. 2018). Anaerobic growth was performed using E agar plates with Anaerocult P (Merck) in a candle jar (Jones 1981) for 3 weeks of incubation. Catalase and oxidase activities were performed according to Maejima et al. (2020) and the production of flexirubin-type pigments was tested according to Reichenbach (Reichenbach 1992). Carbon source utilisation assays were carried out in duplicate using E broth medium along with the Gen III MicroPlate system (Biolog) according to the manufacturer's protocol. Enzyme activities was assayed using the API ZYM (Humble et al. 1977) and API CAMPY (Huysmans et al. 1995) systems (bioMérieux), according to the protocols of the manufacturer. Antibiotic resistances was tested on E agar medium using the disc-diffusion plate method (Bauer et al. 1966).

For chemotaxonomic analysis, strains PWU4^T^, PWU5^T^, PWU20^T^ and PWU37^T^ were grown on M broth medium. Freeze-dried cells were prepared for detection of the major isoprenoid quinones, the polar lipids and the cellular fatty acids of the strains. In brief, 200 ml well-grown cultures were centrifuged at 9000 rpm for 10 min and the pellet was washed three times followed by centrifugation 9000 rpm for 10 min, and then freeze-drying for two days. The isoprenoid quinone was extracted according to Komagata and Suzuki (1988) and analysed further using HPLC (Agilent 1260 Series; Agilent technology USA). The polar lipids were determined by two-dimensional TLC (Lechevalier et al. 1977; Minnikin et al. 1984). The Sherlock Microbial Identification System (MIDI) was used for identifying cellular fatty acids (Sasser 2009).

### 16S rRNA gene sequencing and phylogenetic analysis

The genomic DNA of each strain were extracted using an Invisorb Spin Plant mini kit (Stratec Molecular, Germany). After 5 days incubation at room temperature, cells were harvested from E broth medium and the pellet was further processed through DNA extraction following the manufacturer’s protocol. The 16S rRNA genes was amplified using the bacterial universal primer set 27F and 1492R following the protocol of Mohr et al. (2018). After the PCR products were confirmed on 0.8% agarose gel, they were purified using the NucleoSpin Gel and PCR Clean up Kit (Macherey–Nagel, Düren, Germany). The 16S rRNA gene sequences of strain PWU4^T^, PWU5^T^, PWU20^T^ and PWU37^T^ were determined and compared to sequences from the GenBank7EMBL/DDBJ public database. The Genome to Genome Distance Calculator (GGDC 2.1) web server (Meier-Kolthoff et al. 2013), available at http://ggdc.dsmz.de/ was used to infer the 16S rRNA phylogenetic relationships (Meier-Kolthoff et al. 2014). Briefly, after creating a multiple sequence alignment with MUSCLE (Edgar 2004), maximum likelihood and maximum parsimony trees were inferred with RAxML (Stamatakis 2014) and TNT (Goloboff et al. 2008), respectively. For maximum likelihood, rapid bootstrapping in conjunction with the autoMRE boot stopping criterion (Pattengale et al. 2010) and subsequent search for the best tree was used, while for maximum parsimony, 1000 bootstrapping replicates were used.

### Genome sequence analysis

Draft genome sequences of each of the four strains were determined according to Mohr et al. (2018) and were submitted to the GenBank/EMBL/DDBJ public database. Automated genome annotation was performed using DFAST (Tanizawa et al. 2018). The average nucleotide identity (ANI) values between the genomes of the strains and their close relatives were calculated with the OrthoANIu algorithm using the EZ-Genome web services (Yoon et al. 2017). Digital DNA-DNA hybridization (dDDH) values were calculated using the GGDC 2.1 online service at http://ggdc.dsmz.de/distcalc2.php (Meier-Kolthoff et al. 2014). The phylogenomic analysis, matrix of AAI (Amino Acid Identity) and matrix of POCP (Percentage of Conserved Protein) were carried out using the EDGAR 3.0, a free bioinformatic platform available under https://edgar3.computational.bio.uni-giessen.de where the genome sequence data were uploaded and the output was visualized (Dieckmann et al. 2021).

## Results and discussion

### Morphological, physiological and biochemical analyses

The cells of strains PWU4^T^, PWU5^T^, PWU20^T^ and PWU37^T^ were observed to be straight rods, 2.32–7.62 µm in length, to stain Gram-negative and to form yellow colonies on E medium (Okanya et al. 2011a, b).

Strains PWU4^T^ and PWU20^T^ were found to grow at 21–40 °C (optimum at 28–30 °C), while strains PWU5^T^ and PWU37^T^ grow at 21–34 °C (optimum at 28–34 °C). Moreover, strain PWU4^T^ grows at pH 5.5–8.0 (optimum at pH 7), strain PWU5^T^ grows at pH 6.5–8.5 (optimum at pH 7), strain PWU20^T^ grows at pH 6.5–9.0 (optimum at pH 7) and strain PWU37^T^ grows at pH 5.0–9.5 (optimum at pH 7.4–8.0). The reference strains *Ohtaekwangia koreensis* (3B-2^T^) and *Ohtaekwangia kribbensis* (10AO^T^) (Yoon et al. 2011a), optimally grow at 30 °C in a ranged of 10–39 °C and pH 6.5–7.5 (Table 1). Salt tolerance was tested over the range of 0.2–1.6% NaCl (w/v). Strains PWU4^T^, PWU5^T^, PWU20^T^ and PWU37^T^ can tolerate a concentration up to 0.4, 0.6, 0.8 and 1.0% (w/v), respectively. Reference strains such as members of the genus *Ohtaekwangia,* which were isolated from the marine environment, tolerate a concentration up to 0.2% (w/v) only. Members of the closely related genus *Chryseotaela*, tolerate up to 1.0% of NaCl (w/v) (Maejima et al. 2020) and members of *Chryseolinea* tolerate up to 0.1% of NaCl (w/v) (Kim et al. 2013).

**Table 1:** Major phenotypic characteristics distinguishing strains PWU4^T^, PWU5^T^, PWU20^T^ and PWU37^T^ with members of the genus *Ohtaekwangia*. Strain: 1, PWU4^T^; 2, PWU5^T^; 3, PWU20^T^; 4, PWU37^T^; 5, *Ohtaekwangia koreensis* 3B-2^T^ and 6, *Ohtaekwangia kribbensis* 10AO^T^. +, positive; w, weakly activities, − negative, *taken from Yoon et al. (2011b)

Characteristic	1	2	3	4	5	6
Cell morphology	Rod	Rod	Rod	Rod	Rod	Rod
Cell length (µm)	2.56–6.67	4.37–7.62	2.32–6.41	3.1–6.43	1.0–5.0	1.5–7.5
Temperature range of growth (°C)	21–40	21–34	21–40	21–34	10–39	10–39
Optimal temperature (°C)	30	28	28	34	30	30
pH range of growth	5.5–8.0	6.5–8.5	6.5–9.0	5.0–9.5	5.5–9.0	4.5–9.0
Optimal pH	7	7	7	7.4–8.0	6.5–7.5	6.5–7.5
NaCl tolerance (%NaCl, w/v)	0–0.4	0–0.6	0–0.8	0–1.0	0–0.2	0–0.2
Flexirubin type pigment	−	−	−	−	+	+
Catalase	−	−	−	−	+	+
Oxidase	−	−	−	−	+	+
*Enzyme activity (Api®ZYM, Api®CAMPI)*						
Esterase (C4)	+	w	w	w	−	−
Esterase lipase (C8)	+	w	w	w	−	−
Lipase (C14)	w	w	w	w	−	−
Valine arylamidase	+	+	+	+	+	−
Cystine arylamidase	+	+	+	+	−	−
Trypsin	+	−	w	−	+	−
Chymotrypsin	+	−	+	−	−	−
Phosphatase acid	+	+	+	+	+	−
Naphthol-AS-B1-phosphohydrolase	+	+	+	+	+	−
α-Galactosidase	+	+	+	+	+	−
β-Galactosidase	+	+	+	+	+	−
β-Glucoronidase	w	−	+	−	−	−
α-Glucosidase	+	+	+	+	+	−
β-Glucosidase	+	+	+	+	+	−
α-Mannosidase	+	−	+	−	+	−
α-Fucosidase	+	−	−	+	+	−
Urease	+	−	+	−	−	−
Hippurate	+	−	+	−	+	+
γ-Glutamyl transferase	+	+	+	+	−	−
Reduction of tetrazolium	+	+	+	+	−	−
*Antibiotic resistance*						
Gentamycin (50 µg/ml)	+	+	−	+	−	+
G+C contents (mol%)	50.2	51.6	39.8	53.8	42.8*	44.6*
*Carbon sources utilization*						
D-cellobiose	−	+	−	+	+	+
L-fucose	−	+	−	+	+	+
D-arabitol	−	+	−	+	−	−
D-gluconic acid	+	−	+	−	+	+
Methyl pyruvate	+	−	+	−	−	−
L-malic acid	−	+	−	+	−	+
α-Keto-butyric-acid	−	+	−	+	−	−

Reichenbach, (1992) noted that a few of the members of *Cytophagaceae* are microaerophilic, capnophilic (CO_2_-requiring) or facultatively anaerobic. No growth was observed for any the strains under anaerobic conditions. Catalase and oxidase activities along with flexirubin-type pigments for all the strains were negative, whereas the reference strains are positive for these characteristics (Table 1). Strains PWU4^T^, PWU5^T^, PWU20^T^ and PWU37^T^ are able to metabolise D-galactose and glutamic acid and are not able to use dextrin and N-acetyl-D-glucosamine (Table 1). Strains PWU4^T^, PWU5^T^, PWU20^T^ and PWU37^T^, along with the reference strains (Table 1), are positive for alkaline phosphatase, leucine arylamidase, N-Acetyl-β-glucosamidase, L-arginine arylamidase, L-aspartame arylamidase and alkaline phosphatase. Negative test results were obtained for pyrolidinyl arylamidase, nitrate reduction and H_2_S production. Strains PWU4^T^, PWU5^T^, PWU20^T^ and PWU37^T^ are resistant to polymyxin [50 µg/mL], kanamycin [50 µg/mL], ampicillin [100 µg/mL] and sensitive to chloramphenicol [30 µg/mL].

The major respiratory quinone was identified as menaquinone MK-7 for all the strains, as is also found in the closely related genus *Ohtaekwangia* (Table 1) and almost all of the members of the phylum *Bacteroidetes* (Kim et al. 2013; Maejima et al. 2020; Yoon et al. 2011b) except the family *Flavobacteriaceae*, which have menaquinones of type 6 (MK6) (Albers and Siebers 2014)*.* The major polar lipids of strains PWU4^T^, PWU5^T^, PWU20^T^ and PWU37^T^ were identified as phosphatidylethanolamine and an unidentified polar lipid. The fatty acid profiles of the four strains are shown in Table 2, along with those of the reference strains used in this study. Saturated and monounsaturated fatty acids with iso C_15:0_ and C_16:1_ *ω*7*c* were observed in all the strains including the reference strains. The major fatty acids of strains PWU4^T^, PWU5^T^, PWU20^T^ and PWU37^T^ were identified as C_16:1_ *ω*7*c* (32.5%), C_16:1_ *ω*5*c* (43.8%), iso C_15:0_ (43.6%) and C_16:1_ *ω*5*c* (38.5%), respectively. In contrast to strain PWU4^T^ where C_16:1_ *ω*7*c* was found to be the major fatty acid, this fatty acid was much less abundant in strain PWU37^T^ (9.0%). Moreover, C_16:1_ *ω*5*c* was found to be the major fatty acid in strains PWU5^T^ and PWU37^T^ but not in strains PWU4^T^ and PWU20^T^. Albers and Siebers (2014) highlighted that branched, unsaturated or hydroxyl fatty acids represented the predominant cellular fatty acids in most members of the family *Cytophagaceae.*

**Table 2:** Cellular fatty acid composition of strains PWU4^T^, PWU5^T^, PWU20^T^ and PWU37^T^ compared with members of the genus *Ohtaekwangia.* Strain: 1, PWU4^T^; 2, PWU5^T^; 3, PWU20^T^; 4, PWU37^T^; 5, *Ohtaekwangia koreensis* 3B-2^T^ and 6, *Ohtaekwangia kribbensis* 10AO^T^. Values are percentages of total fatty acids. –, not detected, ECL, equivalent chain-length

Characteristic	1	2	3	4	5	6
*Straight-chain*						
C_14:0_	1.9	–	1.3	1.0	–	–
C1_5:0_	1.8	2.3	–	1.6	–	2.1
C_16:0_	–	2.8	9.7	9.2	–	**22.2**
C_17:0_	–	1.3	–	–	–	–
C_18:0_	2.7	–	1.1	–	–	–
*Branched*						
iso C_13:0_	–	–	–	–	–	–
iso C_14:0_	–	–	–	–	–	1.7
iso C_15:0_	**22.0**	**26.9**	**43.6**	**38.2**	**20.4**	**30.2**
iso C_16:0_	**12.9**	2.2	1.4	–	9.5	4.0
iso C_17:0_	–	–	1.2	1.0	–	7.4
*Unsaturated*						
C_15:1_ *ω*7*c*	1.4	2.8	0.8	–	–	–
C_16:1_ *ω*5*c*	–	**43.8**	–	**38.5**	–	–
C_16:1_ *ω*7*c*	**32.5**	**16.6**	**32.0**	9.0	**55.2**	**27.4**
C_16:1_ *ω*8*c*	–	–	–	0.7	–	–
C_17:1_ *ω*7*c*	–	–	1.0	–	–	–
C_18:1_ *ω*9*c*	8.9	–	3.4	–	–	–
C_18:2_ *ω*6.9*c*	**13.6**	–	4.5	–	–	–
*Hydroxy*						
C_14:0_ 2-OH	1.0	–	–	–	–	–
C_16:0_ 2–OH	1.3	–	–	–	–	–
*Unknown*						
ECL 11.864	–	–	–	–	6.2	3.3
ECL 12.558	–	1.2	–	–	–	–
ECL 16.089	–	–	–	0.7	–	–
ECL 22.207	–	–	–	–	8.7	1.7

### Phylogenetic and genome analysis

**Fig. 1:**
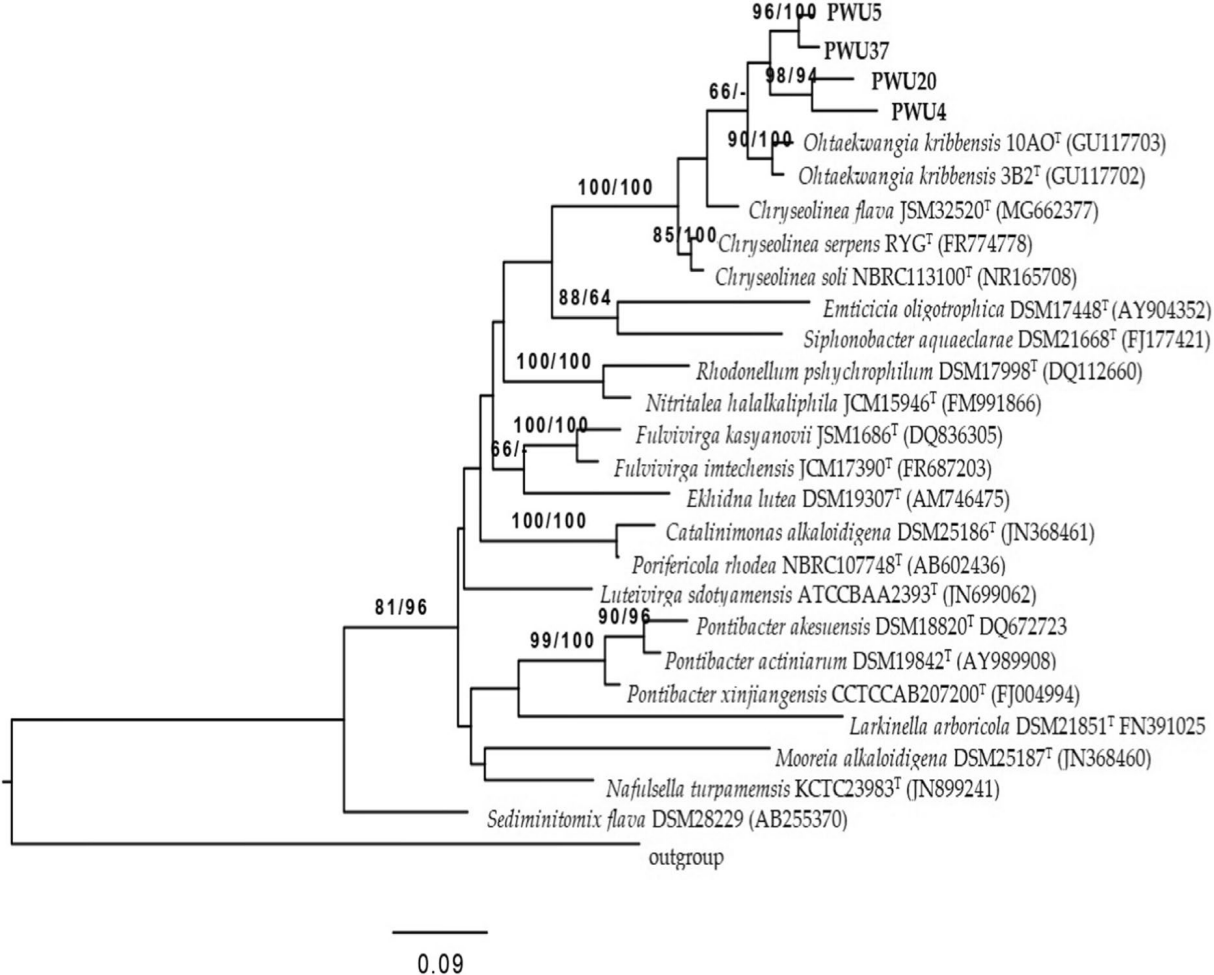
Neighbor-joining phylogenetic tree of partial 16S rRNA gene sequences of strains PWU4^T^, PWU5^T^, PWU20^T^ and PWU37^T^ in comparison with other representatives of the phylum *Bacteriodetes*. Bootstrap value (1000 resampling) at branch nodes (Maximum Parsimony/Maximum-Likelihood). Bar 0.09 substitutions per nucleotide position and *Escherichia coli* DSM 30083 was used as an outgroup

**Fig. 2:**
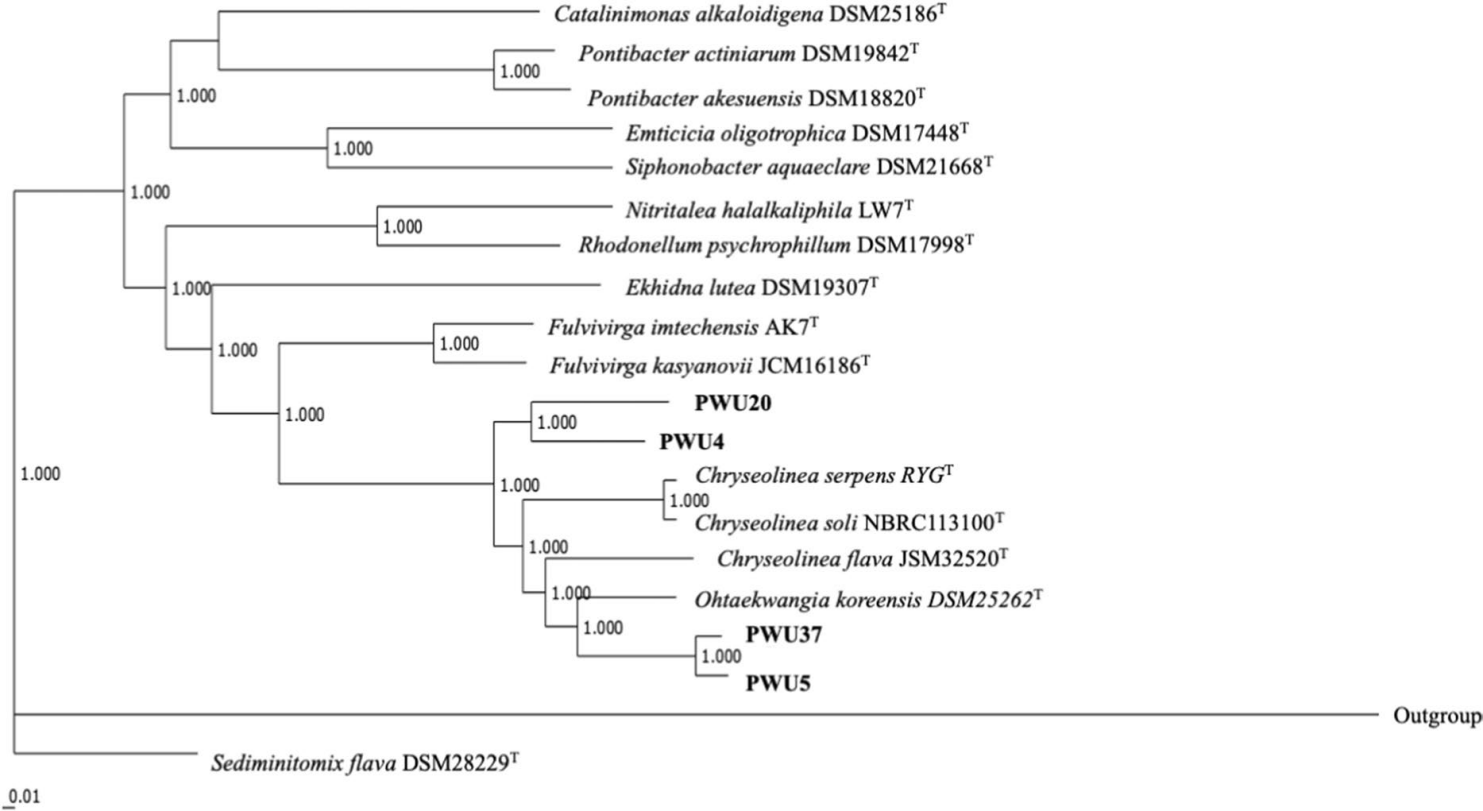
The phylogenomic inference of strains PWU4^T^, PWU5^T^, PWU20^T^ and PWU37^T^ within the EDGAR platform from genome sequences. The tree is built out of core of 309 genes per genome, 6180 in total. The core has 112,516 AA-residues/bp per genome, 2,250,320 in total. The numbers above branches are Shimodaira-Hasagawa (SH) branch support value. *Escherichia coli* DSM 30,083 was used as an outgroup

The phylogenetic tree showed that strains PWU4^T^, PWU5^T^, PWU20^T^ and PWU37^T^ belong to the family *Cytophagaceae* and that they are closely related but distinct from members of the genus *Ohtaekwangia* (Figs. 1, 2). The four strains shared 91.63 to 97.82% 16S rRNA gene sequence similarity with each other (Table 3). The current closest relatives of strain PWU4^T^ are *O. koreensis* 3B-2^T^ (92.1% 16S rRNA gene sequence similarity), *O. kribbensis* 10AO^T^ (92.0%) and *Chryseolinea soli* KIS68-18^T^ (91.0%), whereas the current closest relatives of strain PWU5^T^ are *O. koreensis* 3B-2^T^ (93.6% 16S rRNA gene sequence similarity), *O. kribbensis* 10AO^T^ (93.1%), and *Chryseolinea serpens* RYG^T^ (92.3%). The current closest relatives of strain PWU20^T^ are *O. kribbensis* 10AO^T^ (92.5% 16S rRNA gene sequence similarity), *O. koreensis* 3B-2^T^ (92.0%), and *C. soli* KIS68-18^T^ (90.6%). The current closest relatives of strain PWU37^T^ are *O. kribbensis* 10AO^T^ (93.7% 16S rRNA gene sequence similarity), *O. koreensis* 3B-2^T^ (93.2%) and *C. soli* KIS68-18^T^ (92.0%). Notably, all of these values are below the 94.5% 16S rRNA threshold suggested by Yarza et al. (2014) as being useful for delineating prokaryotic genera.

**Table 3:** Matrix similarity of 16S rRNA gene sequence similarities of strains PWU4^T^, PWU5^T^, PWU20^T^ and PWU37^T^ compared with members of the genus *Ohtaekwangia*

Strain	PWU4^T^	PWU5^T^	PWU20^T^	PWU37^T^
PWU4^T^	100	91.63	93.21	91.98
PWU5^T^	91.63	100	97.82	92.93
PWU20^T^	93.21	97.82	100	93.13
PWU37^T^	91.98	92.93	93.13	100
*O. kribbensis* 10AO^T^	92	93.1	92.5	93.7
*O. koreensis* 3B-2^T^	92.1	93.6	92	93.2

**Fig. 3:**
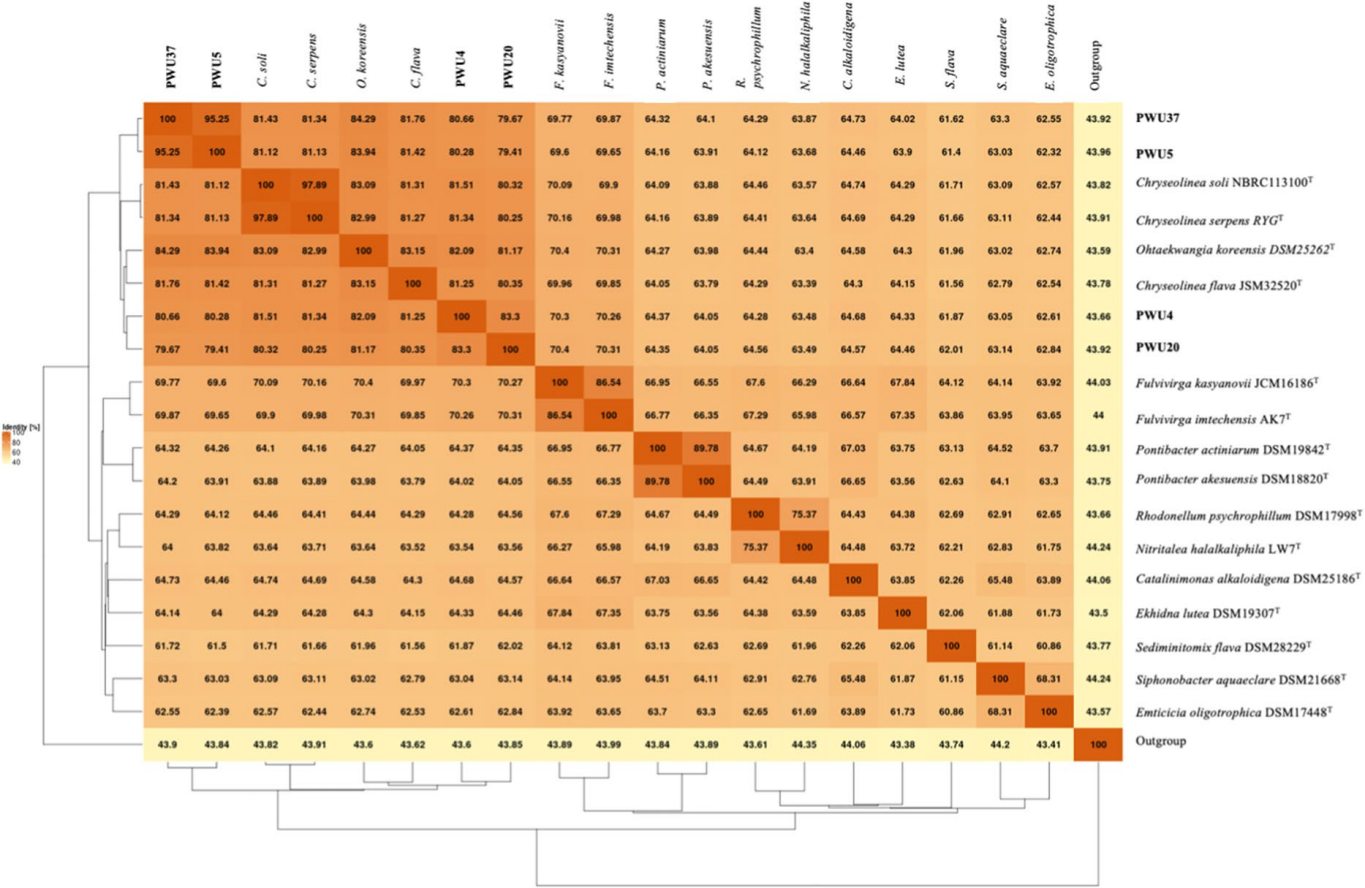
Matrix of Amino Acid Identity (AAI) of strains PWU4^T^, PWU5^T^, PWU20^T^ and PWU37^T^ in comparison with other representatives of the phylum *Bacteriodetes*. Results were obtained using the EDGAR 3.0 platform based on a BLASTN comparison of the genome sequences and *Escherichia coli* DSM 30,083 was used as an outgroup

**Fig. 4:**
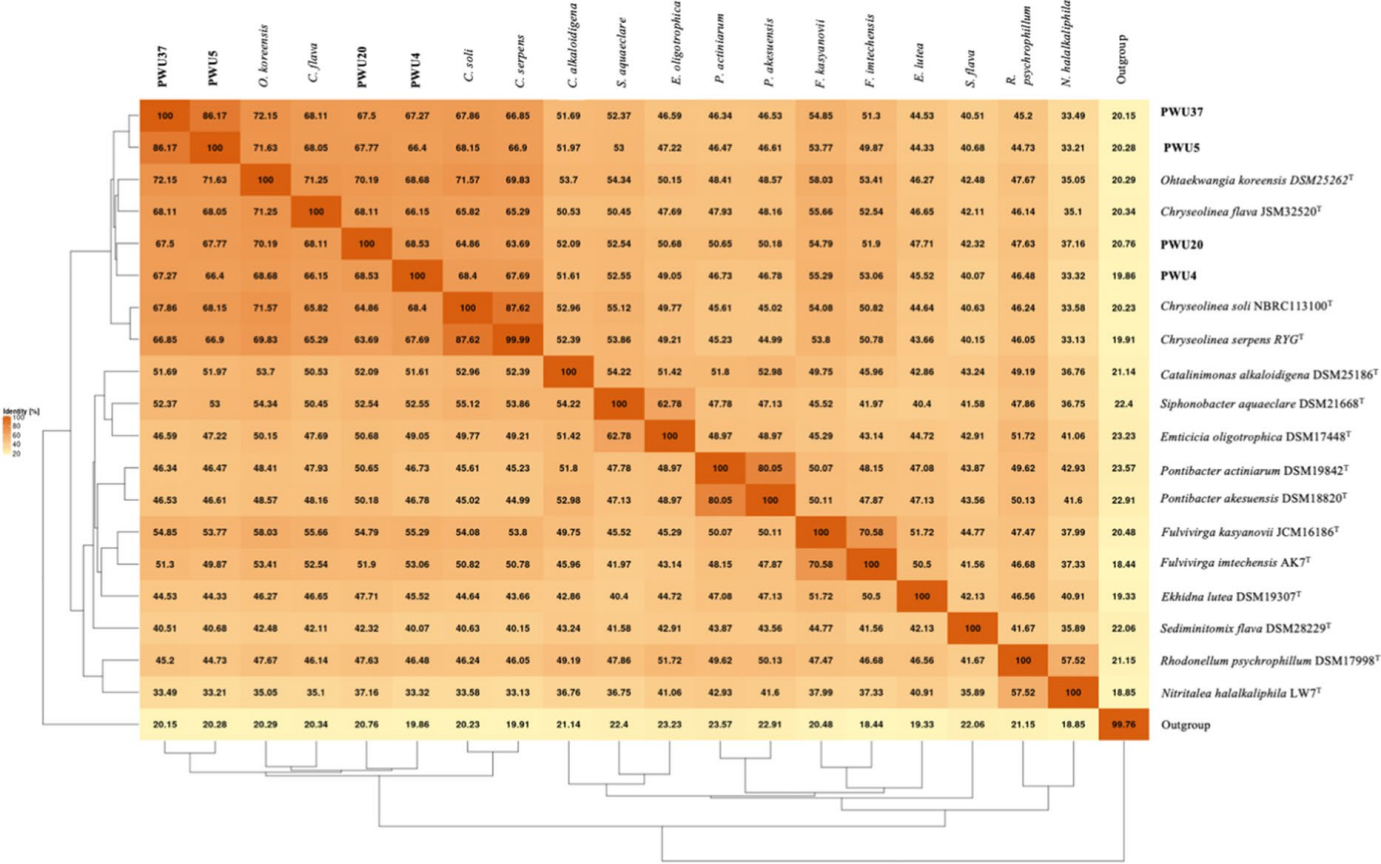
Matrix of POCP (Percentage of Conserved Protein) of strains PWU4^T^, PWU5^T^, PWU20^T^ and PWU37^T^ in comparison with other representatives of the phylum *Bacteriodetes*. Results were obtained using the EDGAR 3.0 platform and *Escherichia coli* DSM 30,083 was used as an outgroup

The phylogenomic tree (Fig. 2), AAI matrix (Fig. 3) and POCP matrix (Fig. 4) confirmed the close relationships of the four strains in the two different genera. Strain PWU5^T^ and PWU37^T^ are grouped in the same genus and strain PWU4^T^ and PWU20^T^ together in a second genus. However, it was noted that between strains PWU4^T^ and PWU20^T^ share only 93.2% 16S rRNA similarity (Table 3) and differed in fatty acid profiles and GC content. At this point we cautiously place these two strains in the same genus based on the 16S rRNA, phylogenomic, AAI and POCP analysis but we noted that further analyses should be conducted.

Interestingly, in contrast to Qin et al. (2014), where the POCP values greater than 50% similarity provided evidence for placing species in the same genus, this threshold does not appropriate within the present study. Similarly other researchers revised the POCP genus threshold with higher values than 50% (Haba et al. 2021; Gupta 2019; Wirth and Whitman 2018). Approximately 65–70% and greater than 80% threshold may be more reasonable values for the genera containing strains PWU4^T^ and PWU20^T^ and strains PWU5^T^ and PWU37^T^, respectively. However, Xu et al. (2020) highlighted that AAI analysis may be more acceptable to distinguish taxa at the genus level than ANI and POCP analysis.

The DNA G + C content of strains PWU4^T^, PWU5^T^, PWU20^T^ and PWU37^T^ were determined to be 50.2 mol%, 51.6 mol%, 39.8 mol% and 53.8 mol%, respectively. The pairwise digital DNA-DNA hybridization (dDDH) revealed values of 13% to 50% and confirmed that all of strains represented distinct new species. Strains PWU4^T^, PWU5^T^, PWU20^T^, and PWU37^T^ shared ANI values of 69.2%, 69.9%, 69.5% and 69.7% with *O. koreensis* 3B-2^T^, respectively (Table 4). The low ANI values below the threshold of 95.0–96.0% (Richter and Rosselló-Móra 2009) confirmed that all four of these strains represented different species to current members of the genus *Ohtaekwangia*.

**Table 4:** Matrix of Average Nucleotide Identity (ANI) of strains PWU4^T^, PWU5^T^, PWU20^T^ and PWU37^T^ compared with *Ohtaekwangia koreensis* 3B-2^T^

Strain	PWU4^T^	PWU5^T^	PWU20^T^	PWU37^T^
PWU4^T^	100	70.22	69.37	70.77
PWU5^T^	70.22	100	67.9	83.85
PWU20^T^	69.37	67.9	100	68.04
PWU37^T^	70.77	83.85	68.04	100
*O. koreensis* 3B-2^T^	69.23	69.92	69.51	69.66

## Taxonomic conclusions

Morphological, biochemical, physiological and phylogenetic characteristics of strains PWU4^T^, PWU5^T^, PWU20^T^ and PWU37^T^ confirmed their position within the family *Cytophagaceae.* However, the obtained genetic data between strains PWU4^T^, PWU5^T^, PWU20^T^, PWU37^T^ and related genera differentiated them from known genera of the family *Cytophagaceae.* Therefore, strains PWU4^T^, PWU5^T^, PWU20^T^ and PWU37^T^ should be classified in two new genera within the family *Cytophagaceae.*

### Description of *Chryseosolibacter* gen. nov.

*Chryseosolibacter* (Chry.se.o.so.li.bac.ter. Gr. masc. adj. chryseos, golden; L. gen. n. soli, soil; L. neut. n. bacter, bacteria; N.L. neut. n. *Chryseosolibacter,* the color of colony bacteria is something like golden soil.

Gram-stain negative, rod-shaped, asporogenous, non-motile, mesophilic, heterophilic and aerobic bacteria. Catalase and oxidase negative. Growth is observed on D-gluconic acid and methyl pyruvate. The major cellular fatty acids are *iso*-C_15:0_ and C_16:1_ *ω*7*c*. The major respiratory quinone is menaquinone-7 (MK-7). The major identified polar lipid is phosphatidylethanolamine. Member of the family *Cytophagaceae.* The type species is *Chryseosolum histidini.*

### Description of Chryseosolibacter histidini sp. nov.

*Chryseosolibacter histidini* (his.ti.di’ni. N.L. gen. n. histidini, of histidine, an amino acid, which can be utilised by this strain).

In addition to the characteristics listed in the genus description, the species has the following characteristics: cells are 2.56–6.67 µm long and appear as single cells. Colonies are irregular and yellow on E agar plates after 5 days of cultivation. Growth occurs between 21 and 40 °C (optimum 30 °C), between pH 5.5 and 8.0 (optimum pH 7) and NaCl tolerance 0.4% (w/v). Positive for phosphatase alkaline, esterase (C4), esterase lipase (C8), leucine arylamidase, valine arylamidase, cystine arylamidase, trypsin, chymotrypsin, phosphatase acid, naphthol-AS-B1-phosphohydrolase, α-galactosidase, β-galactosidase, α-glucosidase, β-glucosidase, N-acetyl-β-glucosamidase, α-mannosidase, α-fucosidase, urease, esterase and alcaline phosphatase. Weak activities of lipase (C14) and β-glucoronidase. No growth is observed on dextrin, D-maltose, D-trehalose, D-cellobiose, gentiobiose, sucrose, D-turanose, stachyose, D-raffinose, α-D-lactose, D-melibiose, N-acetyl-D-glucosamine, N-acetyl-D-galactosamine, α-D-glucose, D-mannose, L-fucose, D-arabitol, glycerol, gelatin, glycyl-L-proline, L-alanine, L-arginine, L-serine, pectin, D-galacturonic acid, L-galactonic acid lactone D-gluconic acid, glucuronic acid, glucuornicamide, mucic acid, D-lactic acid methyl ester, citric acid, D-malic acid, L-malic acid, bromo-succinic acid, tween 40, γ-amino-butryric acid, α-keto-butyric-acid, acetoacetic acid, and acetic acid. The DNA G + C content of the type strain is 50.2 mol%.

The type strain PWU4^T^ (= DSM 111594^T^ = NCCB 100798^T^) was isolated from a soil sample collected in May 1990 at Braunschweig, Germany (52.22090N 10.50902E). The 16S rRNA gene and whole-genome sequences of PWU4^T^ have been deposited in GenBank/EMBL/DDBJ under accession numbers MW182516 and JAHESF000000000, respectively.

### Description of Chryseosolibacter indicus sp. nov.

*Chryseosolum indicus (*in’di.cus. L. neut. adj. indicus*,* India, the origin of the soil sample from which the type strain was isolated*).*

In addition to the characteristics listed in the genus description, the species has the following characteristics: cells are 2.32–6.41 µm long and appear as single cells. Colonies are irregular and yellow on E agar plates after 5 days of cultivation. Growth occurs between 21 and 40 °C (optimum 28 °C), between pH 6.5 and 9.0 (optimum pH 7) and NaCl tolerance below 0.8% (w/v). Positive for phosphatase alkaline, leucine arylamidase, valine arylamidase, cystine arylamidase, chymotrypsin, phosphatase acid, naphthol-AS-B1-phosphohydrolase, α-galactosidase, β-galactosidase, β-glucoronidase, α-glucosidase, β-glucosidase, N-acetyl-β-glucosamidase, α-mannosidase, urease, esterase and alcaline phosphatase. Weak activities of esterase (C4), esterase lipase (C8), lipase (C14), trypsin and no activities for α-fucosidase. No growth is observed on dextrin, D-maltose, D-cellobiose, gentiobiose, N-acetyl-D-glucosamine, N-acetyl-β-D-galactosamine, D-fructose, D-frucose, L-rhamnose, inosine, D-sorbtiol, D-mannitol, D-arabitol, myo-inositol, D-fructose-6-PO_4_, L-alanine, L-arginine, L-histidine, L-galactonic acid lactone D-gluconic acid, p-hydroxy-phenylacetic acid, L-malic acid, bromo-succinic acid, tween 40, α-keto-butyric-acid, propionic acid, and acetic acid. The DNA G + C content of the type strain is 39.8 mol %.

The type strain PWU20^T^ (= DSM 111597^T^ = NCCB 100800^T^) was isolated from a soil sample collected in May 1989 at Lucknow, Uttar Pradesh, India (26.8684N 80.90979E). The 16S rRNA gene and whole-genome sequences of strain PWU20^T^ have been deposited in GenBank/EMBL/DDBJ under accession numbers MW182517 and JAHESD000000000, respectively.

### Description of *Dawidia* gen. nov.

*Dawidia* (Da.wi’di.a N.L. fem. n. Dawidia, named in honor of the German microbiologist Dr. Wolfgang Dawid, author of *Experimentelle Mikrobiologie*.)

Gram-stain negative, rod-shaped, asporogenous, non-motile, mesophilic, heterophilic and aerobic bacteria. Catalase and oxidase-negative. Growth is observed on D-cellobiose, L-fucose, D-arabitol, L-malic acid, and α-keto-butyric-acid. The major cellular fatty acids are *iso*-C_15:0_ and C_16:1_ *ω*5*c*. The major respiratory quinone is menaquinone-7 (MK-7). The major identified polar lipids is phosphatidylethanolamine (PE). Member of the family *Cytophagaceae*. The type species is *Dawidia cretensis.*

### Description of *Dawidia cretensis s*p. nov.

*Dawidia cretensis* (cre.ten`sis. L. fem. adj. cretensis, Cretan, the source of the sample from the type strain was isolated).

In addition to the characteristics listed in the genus description, the species has the following characteristics: cells are 4.37–7.62 µm long and appear as single cells. Colonies are irregular and yellow on E agar after 5 days of cultivation. Growth occurs between 21 and 34 °C (optimum 28 °C), between pH 6.5 and 8.5 (optimum pH 7) and NaCl tolerance below 0.6% (w/v). Positive for phosphatase alkaline, leucine arylamidase, valine arylamidase, cystine arylamidase, phosphatase acid, naphthol-AS-B1-phosphohydrolase, α-galactosidase, β-galactosidase, α-glucosidase, β-glucosidase, N-acetyl-β-glucosamidase, esterase and alkaline phosphatase. Weak activities of esterase (C4), esterase lipase (C8), lipase (C14) and negative for trypsin, chymotrypsin, β-glucoronidase, α-mannosidase, α-fucosidase and urease activities. No growth is observed on dextrin, D-turanose, α-D-lactose, N-acetyl-D-glucosamine, N-acetyl-D-galactosamine, L-arginine, L-histidine, L-pyroglutamic acid, D-gluconic Acid, glucuronic acid, glucuornicamide, mucic acid, quinic acid, D-saccharic acid, methyl pyruvate, citric acid, γ-amino-butryric acid, and β-hydroxy-D,L-butyric acid. The DNA G + C content of the type strain is 51.6 mol %.

The type strain PWU5^T^ (= DSM 111596^T^ = NCCB 100799^T^) was isolated from sheep faeces with plant residues collected in July 1988 on the Island of Crete (35.2463N 25.09705E). The 16S rRNA gene and whole-genome sequences of strain PWU5^T^ have been deposited in GenBank/EMBL/DDBJ under accession numbers MW182518 and JAHESE000000000, respectively.

### Description of *Dawidia soli* sp. nov.

*Dawidia soli* (so.li. L. gen. n. soli, of soil, the source of the type strain).

In addition to the characteristics listed in the genus description, the species has the following characteristics: cells are 3.1–6.43 µm long and appear as single cells. Colonies are irregular and yellow on E agar plate after 5 days of cultivation. Growth occurs between 21 and 34 °C (optimum 34 °C), between pH 5.0 and 9.5 (optimum pH 7.4 until 8.0) and NaCl tolerance below 1.0% (w/v). Positive for phosphatase alkaline, leucine arylamidase, valine arylamidase, cystine arylamidase, phosphatase acid, naphthol-AS-B1-phosphohydrolase, α-galactosidase, β-galactosidase, α-glucosidase, β-glucosidase, N-acetyl-β-glucosamidase, α-fucosidase, esterase and alkaline phosphatase. Weak activities of esterase (C4), esterase lipase (C8), lipase (C14) and no activities for trypsin, chymotrypsin, β-glucoronidase, α-mannosidase, and urease. No growth is observed on dextrin, D-maltose, gentiobiose, D-turanose, D-raffinose, α-D-lactose, D-melibiose, D-salicin, N-acetyl-D-glucosamine, N-acetyl-β-D-galactosamine, N-acetyl-D-galactosamine, α-D-glucose, D-fructose, D-sorbitol, myo-inositol, D-glucose-6-PO4, D-fructose-6-PO_4_, D-aspartic acid, D-serine, gelatin, L-alanine, L-arginine, L-aspartic Acid, L-histidine, pectin, L-galactonic acid lactone D-gluconic acid, D-gluconic acid, glucuornicamide, mucic acid, p-hydroxy-phenylacetic acid, methyl pyruvate, D-lactic acid methyl ester, L-lactic acid, citric acid, α-keto-glutaric acid, D-malic acid, bromo-succinic acid, tween 40, α-hydroxy-butyric acid, β-hydroxy-D,L-butyric acid, acetoacetic acid, propionic acid, acetic acid and formic acid. The DNA G + C content of the type strain is 53.8 mol %.

The type strain PWU37^T^ (= DSM 111595^T^ = NCCB 100801^T^) was isolated from a soil sample collected in September 1991 at Braunschweig (52.21501N 10.53329E). The 16S rRNA gene and whole-genome sequences of strain PWU37^T^ have been deposited in GenBank/EMBL/DDBJ under accession numbers MW182519 and JAHESC000000000, respectively.

## Abbreviations

DSMZ: *Deutsche Sammlung von Mikroorganismen und Zellkulturen GmbH*
GGDC: Genome to Genome Distance Calculator
G + C: Guanine + Cytosine
MEGA X: Molecular Evolutionary Genetics Analysis
NCBI: National Center for Biotechnology Information
PAUP*: Phylogenetic Analysis Using Parsimony
PCR: Polymerase Chain Reaction
RAxML: Randomized Accelerated Maximum Likelihood
RDP: Ribosomal Database Project
RNA: Ribonucleic Acid
rRNA: Ribosomal Ribonucleic Acid
TNT: Tree analysis using New Technology
